# Commentary: How Bayes factors change scientific practice

**DOI:** 10.3389/fpsyg.2016.01504

**Published:** 2016-09-30

**Authors:** Jose D. Perezgonzalez

**Affiliations:** Business School, Massey UniversityPalmerston North, New Zealand

**Keywords:** Bayes factors, significance testing, acceptance testing, meta-analysis, replication, Fisher, Neyman–Pearson, Jeffreys

Dienes's (2016) article is one of the contributions to the special issue “Bayes factors for testing hypotheses in psychological research…” being published by the *Journal of Mathematical Psychology*. It is the article most accessible to non-Bayesians, offering a good understanding of Jeffreys's data testing approach (Bayes factors) with little in the form of mathematical expressions.

Dienes's main argument is one of best-fit-for-purpose: the Bayes factor pits the probability of the data under one hypothesis against that under another on equal ground, providing a symmetric assessment—the data may favor either hypothesis, or neither—as a continuous measure of evidence in the form of odds. For Dienes, Jeffreys's (1961) approach is, if not perfect, at least superior to those of Fisher's (1954) tests of significance and of Neyman and Pearson's (1933) tests of acceptance. Unlike Bayes factors, Fisher's approach only tests data under a null hypothesis so that the resulting *p*-value is asymmetric, capable of providing evidence against such hypothesis but not in its favor. Neyman–Pearson's approach, on the other hand, uses two hypotheses and allows some ground for asserting either if the power of the test is adequate; however, it is not evidential insofar it has little use for sample statistics such as *p*-values and *post-hoc* power. Therefore, not only the use of Bayes factors is a much better approach for testing research data but such use will also “help solve some…of the problems leading to the credibility crisis” (p. ii) posed by the latter two approaches.

One concern I have with Dienes's article is its “one-size-fits-all” philosophy. Allow me to argue the point using non-research affairs, which seem more relatable. Most (if not all) of us have certainly been in the position of having to choose between valuable alternatives, pitting one against the other and selecting that which came on top. Such positions may range from the serious— “Which cancer treatment to choose, radiotherapy or surgery?”—to the rather banal—“Coffee or tea?” However, there are times when decisions do not need, nor benefit from, such pitting among defined alternatives. “Do I have a temperature?” is a question that calls for assessing data against a known cut-off that rejects the normal hypothesis in favor of the sick hypothesis without the need to test the latter. There are also many times when decisions are based on assessing just a single model in reference to standards of its own and not in relation to alternative hypotheses, such as deciding whether we are enjoying our lunch or whether we are happy with our lives.

Furthermore, there are occasions in which any of the three methods may be used depending on how the situation comes to us. For it is possible for the same person to decide to divorce if a comparatively better person comes along one day, as it is for him or her to divorce only after high thresholds of regret between omission and commission have been breached in a long-run of mulling over the possibilities, as it is to divorce for reasons other than the existence of alternatives (e.g., because the person has just been abused by her or his current partner).

Most research in psychology fit well the aims of a test of significance, especially in regards to null hypotheses been uninteresting models which serve only the purpose of offering an exact distribution against which to test the research data at hand. For if one is just interested in the significance of a treatment (as in its practical importance using small samples, Perezgonzalez, 2015a), what is to be gained from supporting the null? The situation would certainly be different if one were interested in both, for example because ineffective treatments could be used as placebo in future research projects or because the null represents a general law (Jeffreys, 1961; also Robert, 2016). In the latter cases, a null model is equally interesting and Bayes factors relevant. Thus, I find it naive that a single approach is still proposed as the one and only tool for testing data. It is true that a research question may be adapted to suit a particular tool but this does not guarantee that such tool will address the research question correctly.

A second concern is the reification of Bayes factors as the solution to the credibility crisis. The thing is, it is not just a single research which will solve the crisis but replication (also, R-Index, 2015, 2016). Interestingly enough, Fisher advocated the accumulation of evidence (thus, hinting to cumulative meta-analysis, e.g., Braver et al., 2014), while Neyman–Pearson's approach calls for an acceptable proportion of direct replications in long-run sequences (Perezgonzalez, 2015b). Jeffreys's approach sits within the updating philosophy of Bayes–Laplace's formula, yet replicability is rarely emphasized by Bayes factors proponents (e.g., Dienes, 2016; Ly et al., 2016; Morey et al., 2016).

And this latter concern brings me back to the title of the special issue because a Bayes factor is that part of the Bayes-Laplace formula that deals with the probability of the data under each hypothesis excluding the prior probabilities of the hypotheses themselves. This puts Bayes factors at the same level as Fisher's *p*-values and Neyman–Pearson's error decisions (which may partly explain why “in spite of the difference in principle between my tests and those based on the *p* integrals…it appears that there is no much difference in the practical recommendations,” Jeffreys, 1961, p. 435). Bayes factors proponents do not usually address how replicability is to be managed—and those who do mostly rely on frequentist statistics by tallying the Bayes factors of individual studies (e.g., Wagenmakers et al., 2011), calculating the Bayes factors of frequentist meta-analyses (e.g., Etz, 2015), or calculating the Bayes factors of test statistics (e.g., Rouder and Morey, 2011). Thus, I also find it naive to assume that Bayes factors, with no clear replicability mechanism attached to them, are the ones to resolve the credibility crisis in psychology.

## Author contributions

The author confirms being the sole contributor of this work and approved it for publication.

### Conflict of interest statement

The author declares that the research was conducted in the absence of any commercial or financial relationships that could be construed as a potential conflict of interest.
